# Harnessing peripheral DNA methylation differences in the Alzheimer’s Disease Neuroimaging Initiative (ADNI) to reveal novel biomarkers of disease

**DOI:** 10.1186/s13148-020-00864-y

**Published:** 2020-06-15

**Authors:** Aparna Vasanthakumar, Justin W. Davis, Kenneth Idler, Jeffrey F. Waring, Elizabeth Asque, Bridget Riley-Gillis, Shaun Grosskurth, Gyan Srivastava, Sungeun Kim, Kwangsik Nho, Kelly N. H. Nudelman, Kelley Faber, Yu Sun, Tatiana M. Foroud, Karol Estrada, Liana G. Apostolova, Qingqin S. Li, Andrew J. Saykin, for the Alzheimer’s Disease Neuroimaging Initiative (ADNI)

**Affiliations:** 1 Genomics Research Center, AbbVie, North Chicago, IL USA; 2 Exploratory Statistics, AbbVie, North Chicago, IL USA; 3 Center for Neuroimaging, Department of Radiology and Imaging Sciences, Indiana University School of Medicine, Indianapolis, IN USA; 4 National Centralized Repository for Alzheimer’s Disease and Related Dementias, Indiana University School of Medicine, Indianapolis, IN USA; 5 Neuroscience Therapeutic Area, Janssen Research & Development, Pennington, NJ 08534 USA; 6 Research Information Technology, Janssen Research & Development, Pennington, NJ 08534 USA; 7 Biogen, Cambridge, MA 02142 USA; 8 Electrical and Computer Engineering, State University of New York, Oswego, NY 13126 USA; 9 Currently at Biomarin Pharmaceuticals, Novato, CA 94949 USA

**Keywords:** Alzheimer’s disease, Peripheral blood, Biomarker, DNA methylation, ADNI

## Abstract

**Background:**

Alzheimer’s disease (AD) is a chronic progressive neurodegenerative disease impacting an estimated 44 million adults worldwide. The causal pathology of AD (accumulation of amyloid-beta and tau), precedes hallmark symptoms of dementia by more than a decade, necessitating development of early diagnostic markers of disease onset, particularly for new drugs that aim to modify disease processes. To evaluate differentially methylated positions (DMPs) as novel blood-based biomarkers of AD, we used a subset of 653 individuals with peripheral blood (PB) samples in the Alzheimer’s disease Neuroimaging Initiative (ADNI) consortium. The selected cohort of AD, mild cognitive impairment (MCI), and age-matched healthy controls (CN) all had imaging, genetics, transcriptomics, cerebrospinal protein markers, and comprehensive clinical records, providing a rich resource of concurrent multi-omics and phenotypic information on a well-phenotyped subset of ADNI participants.

**Results:**

In this manuscript, we report cross-diagnosis differential peripheral DNA methylation in a cohort of AD, MCI, and age-matched CN individuals with longitudinal DNA methylation measurements. Epigenome-wide association studies (EWAS) were performed using a mixed model with repeated measures over time with a *P* value cutoff of 1 × 10^−5^ to test contrasts of pairwise differential peripheral methylation in AD vs CN, AD vs MCI, and MCI vs CN. The most highly significant differentially methylated loci also tracked with Mini Mental State Examination (MMSE) scores. Differentially methylated loci were enriched near brain and neurodegeneration-related genes (e.g., *BDNF, BIN1, APOC1*) validated using the genotype tissue expression project portal (GTex).

**Conclusions:**

Our work shows that peripheral differential methylation between age-matched subjects with AD relative to healthy controls will provide opportunities to further investigate and validate differential methylation as a surrogate of disease. Given the inaccessibility of brain tissue, the PB-associated methylation marks may help identify the stage of disease and progression phenotype, information that would be central to bringing forward successful drugs for AD.

## Background

Nearly 44 million people worldwide have Alzheimer’s disease (AD) or a related dementia, with global costs of the disease estimated to be approximately $600 billion in 2016 and steadily increasing as the population ages, making it a major public health issue [1, 2]. The hallmark symptoms of AD include memory impairment and cognitive decline, both of which largely drive clinical diagnosis. Existing therapies do not treat the underlying cause of the disease, and only temporarily help relieve memory and cognitive problems. There are several drugs currently under development which aim to modify the disease process; however, there still exists a lack of understanding regarding the molecular mechanisms underlying the disease, thus making it challenging to identify new targets for therapy. Accurate diagnosis of prodromal AD is essential to starting treatments at the right time, and in treating the disease more effectively [3]. The identification of a robust, prodromal, and easily accessible biomarker has been of major interest in the field.

The Alzheimer’s Disease Neuroimaging Initiative (ADNI) was launched in 2003 with the goal to establish an optimal panel of clinical assessments: imaging measures (MRI, PET) and biomarkers from blood and cerebrospinal fluid (CSF) to direct clinical trial design for AD drugs [4, 5]. We sought to use this resource to determine if epigenetic markers in PB could serve as biomarkers of AD.

Epigenetic modifications are inheritable and dynamic, and may lead to the regulation of gene expression via modifications to the cytosine residues and/or proteins associated with nucleosome assembly and function [6]. Methylation of the DNA cytosine bases has been studied for several decades and studies have associated methylation at promoter regions with repression of gene expression [7]. DNA methylation changes as a result of mutations in the DNA methyltransferase-1-enzyme have been shown to be associated with several neuronal diseases including hereditary sensory and autonomic neuropathy-1, in which patients display disrupted methylation patterns potentially contributing to neurodegeneration [8]. De novo mutations of MeCP2, a methyl CpG-binding protein, are linked to Rett syndrome, a progressive neurodevelopmental disorder [9]. Other epigenetic mechanisms link exposures during the course of life such as nutrition, chemical and emotional environments, pregnancy conditions, drug intake, and social status to long-term health of the individual [10, 11]. These observations and others support the significance of DNA methylation and associated machinery in the temporal control of neural stem cell differentiation, neurodevelopment, and neurodegeneration.

Several studies have observed widespread alterations in DNA cytosine methylation patterns both at the global level as well as at the individual loci in AD brains (reviewed in [12–15]). In 2014, two seminal papers identified DNA methylation patterns that characterize AD brains and correlate with progression as defined by their Braak stages [16, 17]. Given that observed differences in DNA methylation levels across tissues are stable in a healthy individual, and may be exploited to determine early changes associated with disease processes [18, 19], we sought to understand patterns of peripheral blood DNA methylation in the ADNI cohort. Our objectives from this study were to (1) generate a public resource for peripheral DNA methylation marks in a cohort of cognitively normal, MCI, and AD patients; (2) to identify cross-sectional differences in peripheral blood DNA methylation associated with mild cognitive impairment (MCI) and AD patients relative to cognitively normal controls (CN); and (3) identify novel non-invasive disease biomarkers. This information would also help identify subjects who are more susceptible to disease progression. Our goal is to gain a broader understanding of how peripheral DNA methylation differences correlate with the diagnosis of and progression of Alzheimer’s disease and to enable the research and clinical community to leverage these results to assess the potential for use of methylation changes as pharmacodynamic or disease modifying biomarkers.

## Results

### Making available a robust resource for DNA methylation differences in the peripheral blood of Alzheimer’s disease patients

A total of 1920 samples from 653 individual subjects (CN, MCI, AD) were analyzed using the Illumina EPIC arrays (Table 1). Two experimental factors were considered for patient selection: (1) time- our ability to capture the longitudinal aspect of the study (patients with samples at two or more visits), and (2) diagnosis and its time-varying nature (patients converting from CN to MCI, CN to AD, or MCI to AD). Details of patient selection are included in the “Methods” section. The current study focuses on differential methylation analysis of subjects based on diagnosis. One hundred and ninety-nine duplicates and a single triplicate were included amongst the samples that were run on the EPIC arrays for technical replication but are not used in the final analysis here.

**Table 1:** ADNI patient cohort selected for DNA methylation analysis and used for final analysis after normalization and quality control

Starting Patient Cohort (N=653; 1720 DNA samples)					
Diagnosis Groups	Age in years Mean (SD)		Number of Males (%)		Number APOE e4 positive (%)
Cognitively Normal (n=223)	76.23 (6.7)		112 (50.2%)		58 (26%)
Mild Cognitive Impairment (n=336)	72.58 (7.82)		189 (56.3%)		153 (46%)
Alzheimer's Disease (n=94)	77.19 (7.69)		60 (63.8%)		64 (68%)
After Normalization , Quality Control and Removal of Replicates (total 1707)					
Diagnosis at 1^st^ Visit	Number at Visit 1	Number at Visit 2	Number at Visit 3	Number at Visit 4	Number at Visit 5
Cognitively Normal	220	200	162	15	4
Mild Cognitive Impairment	333	312	235	23	4
Alzheimer’s Disease	94	53	51	-	-

### Distribution of differentially methylated positions (DMPs) is consistent across each cross-diagnosis comparison

**Fig. 1:**
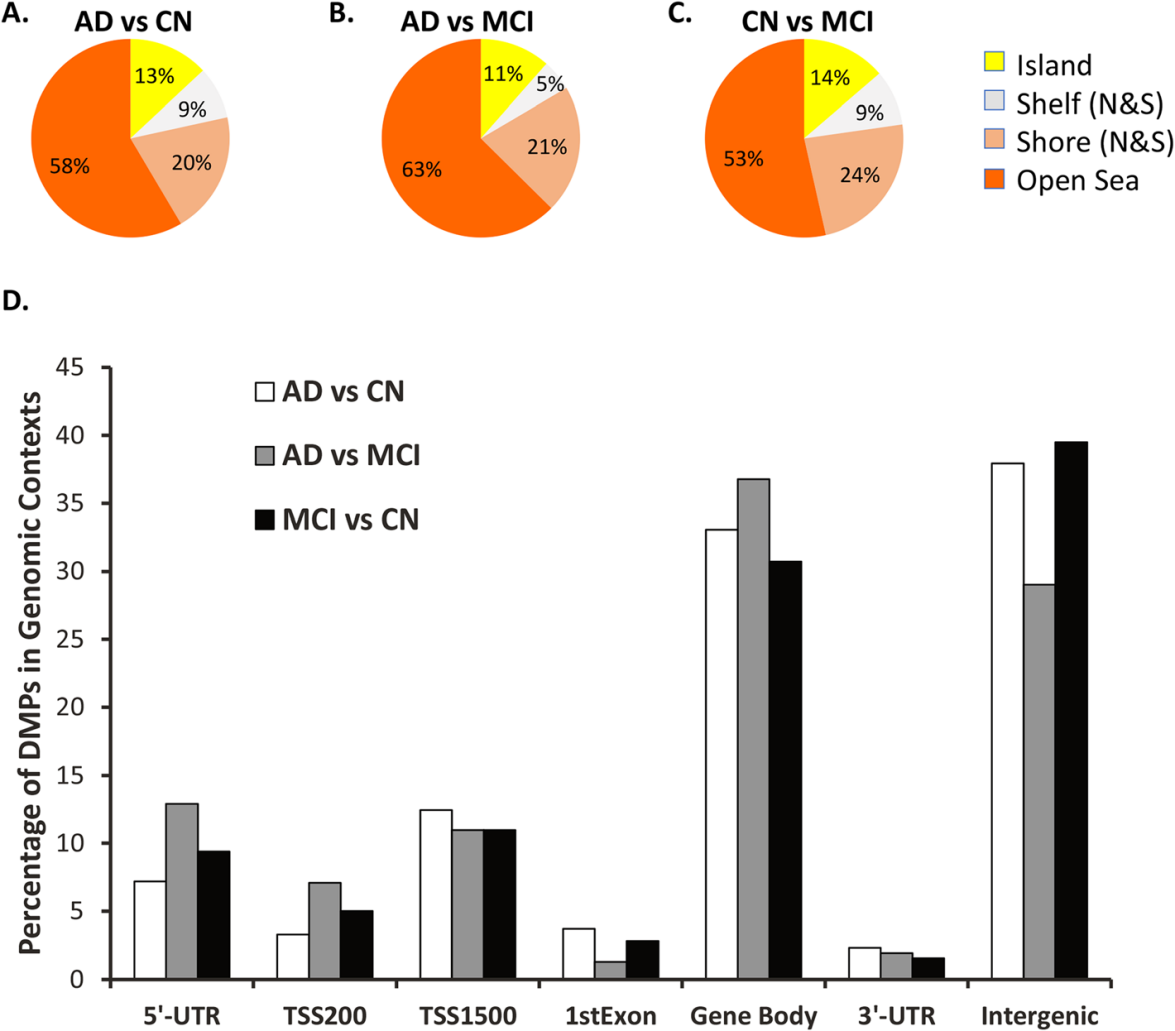
Distribution of cross-diagnosis differential DNA methylation marks across the genome. **a**–**c** Distribution of differential DNA methylation marks relative to the CpG island. Islands are denoted by yellow, shelves (regions 2–4 kb from CpG Islands) by purple, shores (regions 0–2 kb from CpG Islands) by blue and the open seas (genomic loci that fall outside of the islands, shelves, and shores) by orange. Percentages are calculated as percent total number of hits. **d** Distribution of differential DNA methylation marks across different genomic loci. Annotations of the locations are obtained from Illumina EPIC manifests: TSS1500 = within 1500 bp of transcription start site (TSS); TSS200 = within 200 bp of TSS

After extensive quality control evaluation to filter poor probes and low-quality samples, the data were normalized and M-values (i.e., the logit of the beta values) were used for all further analyses. We analyzed differential DNA methylation across diagnosis groups using a mixed model with a random effect to account for within-subject dependency as detailed in the methods section. This allowed us to include all available time points for all subjects. The model included covariates to adjust for age at diagnosis, sex, educational attainment, and peripheral blood cell composition, and this yielded 260, 91, and 137 DMPs, respectively, for the three clinical phenotypic comparisons: AD vs. CN, AD vs. MCI, and MCI vs. CN, with a *p* value threshold of 1 × 10^−5^ (Table S2). The majority of the DMPs were clustered within the open seas (genomic loci that fall outside of the CpG islands), and the adjacent shores (regions 0–2 kb from CpG islands), and shelves (regions 2–4 kb from CpG islands) (Fig. 1a–c). The relative levels of enrichment of specific genomic regions (e.g., gene body, 5′-UTR) within the DMP list from three comparisons were similar and did not show significant differences (Fig. 1d).

### DMPs from each pairwise comparisons are enriched for brain-related pathways

**Fig. 2:**
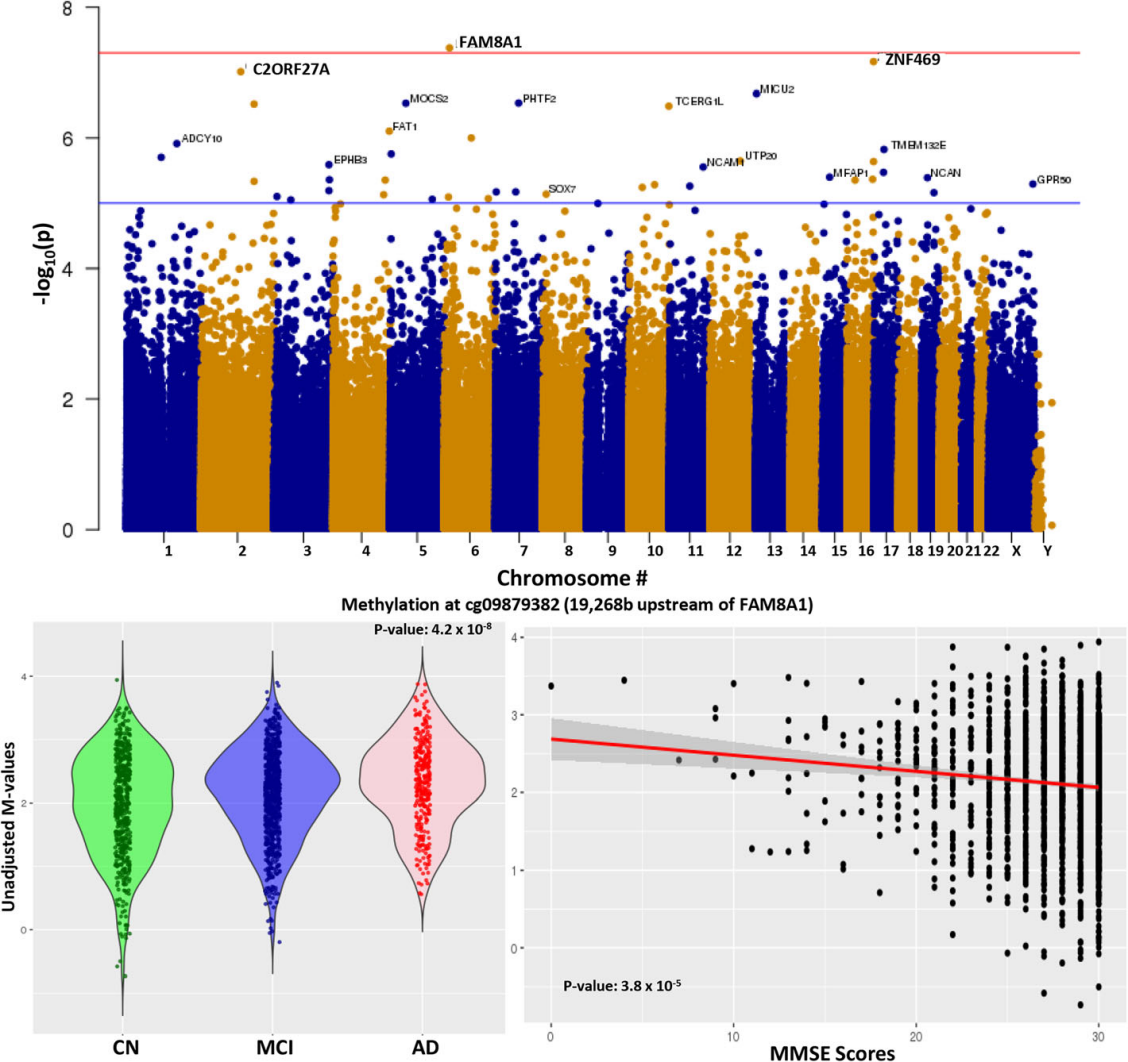
Comparison of DNA methylation in AD (Alzheimer’s disease) vs CN (cognitively normal). **a** Manhattan plot showing the top hits in the AD vs CN comparison. The blue line indicates *p* value threshold of 1 × 10^−5^ and the red line indicates *p* value threshold of 1 × 10^−7^. **b** Distribution of unadjusted *M* values in *FAM8A1*, the top DMP across CN (green), MCI (blue), and AD (red). Violin plots outline the spread of the data. **c** Correlation of MMSE scores with differential methylation at the *FAM8A1* locus

There were 42 DMPs that cleared the p-value of 1 × 10^−5^ in the AD vs CN comparison (Fig. 2a). The DMP that was most significantly associated with AD relative to CN was annotated to *FAM8A1*, which encodes a protein that is associated with endoplasmic reticulum-associated degradation of proteins with roles in Alzheimer’s disease pathogenesis (Fig. 2b). Additionally, when we interrogated the genes located closest to the top DMPs using Tissue Specific Expression Analysis (TSEA), a web-based tool designed to look for tissue-specific expression patterns across 25 different tissue types via GTex Data [20, 21], we observed enrichment for brain-specific genes (*P*_adj_-val = 9 × 10^−4^) (Figure S3A, Table S3). Other tissues that showed enrichment for the AD vs. CN comparison included: pituitary (*P*_adj._-value = 0.016) and uterus (*P*_adj._-value = 9 × 10^−4^). We measured the correlation of observed differential DNA methylation with a cognitive score, MMSE (the mini-mental status examination) and found a significant (*p* value = 3.8 × 10^−5^) correlation of MMSE, with DNA methylation differences at this locus (Fig. 2c). We tested the enrichment of neural gene expression in parallel using gene ontology analysis, which identified neurogenesis and neuronal differentiation as some of the most highly enriched pathways in the AD vs. CN annotated DMPs (Table 2).

**Table 2:** Gene ontology analysis of genes within 50 kb of differentially methylated positions from each cross-diagnosis comparison

Gene Set Name	# Genes in Gene Set (K)	# Genes in overlap (k)	k/K	FDR q-value
Cross-Diagnosis Comparison of AD vs CN at All Visits				
BENPORATH_SUZ12_TARGETS	1038	37	0.0356	1.98E-22
BENPORATH_ES_WITH_H3K27ME3	1118	38	0.034	2.60E-22
BENPORATH_EED_TARGETS	1062	37	0.0348	4.29E-22
**GO_NEURON_DIFFERENTIATION**	**874**	**29**	**0.0332**	**6.78E-17**
**GO_NEUROGENESIS**	**1402**	**35**	**0.025**	**2.00E-16**
BENPORATH_PRC2_TARGETS	652	25	0.0383	4.04E-16
GO_CELL_DEVELOPMENT	1426	33	0.0231	1.36E-14
MIKKELSEN_NPC_HCP_WITH_H3K27ME3	341	18	0.0528	2.97E-14
MEISSNER_BRAIN_HCP_WITH_H3K4ME3_AND_H3K27ME3	1069	28	0.0262	8.47E-14
MIKKELSEN_MEF_HCP_WITH_H3K27ME3	590	20	0.0339	3.87E-12
Cross-Diagnosis Comparison of MCI vs CN at All Visits				
**GO_NEUROGENESIS**	**1402**	**18**	**0.0128**	**1.35E-09**
**GO_CELL_PROJECTION**	**1786**	**20**	**0.0112**	**1.52E-09**
MEISSNER_NPC_HCP_WITH_H3_UNMETHYLATED	536	12	0.0224	2.32E-09
BENPORATH_SUZ12_TARGETS	1038	15	0.0145	7.80E-09
**MEISSNER_BRAIN_HCP_WITH_H3K4ME3_AND_H3K27ME3**	**1069**	**15**	**0.014**	**1.15E-08**
BENPORATH_ES_WITH_H3K27ME3	1118	15	0.0134	2.08E-08
GO_CELL_DEVELOPMENT	1426	16	0.0112	7.88E-08
BENPORATH_EED_TARGETS	1062	14	0.0132	7.96E-08
**GO_CELL_PROJECTION_PART**	**946**	**13**	**0.0137**	**1.50E-07**
GSE40443_INDUCED_VS_TOTAL_TREG_UP	200	7	0.035	3.03E-07
Cross-Diagnosis Comparison of AD vs MCI at All Visits				
GO_INTRINSIC_COMPONENT_OF_PLASMA_MEMBRANE	1649	15	0.0091	7.16E-08
GO_CELL_SURFACE	757	10	0.0132	4.97E-07
GRYDER_PAX3FOXO1_ENHANCERS_IN_TADS	975	11	0.0113	6.04E-07
**GO_CELL_PROJECTION**	**1786**	**14**	**0.0078**	**1.22E-06**
BIOCARTA_LAIR_PATHWAY	17	3	0.1765	2.91E-06
GO_ALPHA_ACTININ_BINDING	21	3	0.1429	5.66E-06
**BLALOCK_ALZHEIMERS_DISEASE_DN**	**1237**	**11**	**0.0089**	**5.94E-06**
**GO_NEURON_PART**	**1265**	**11**	**0.0087**	**7.34E-06**
GO_REGULATION_OF_TRANSPORT	1804	13	0.0072	7.65E-06

**Fig. 3:**
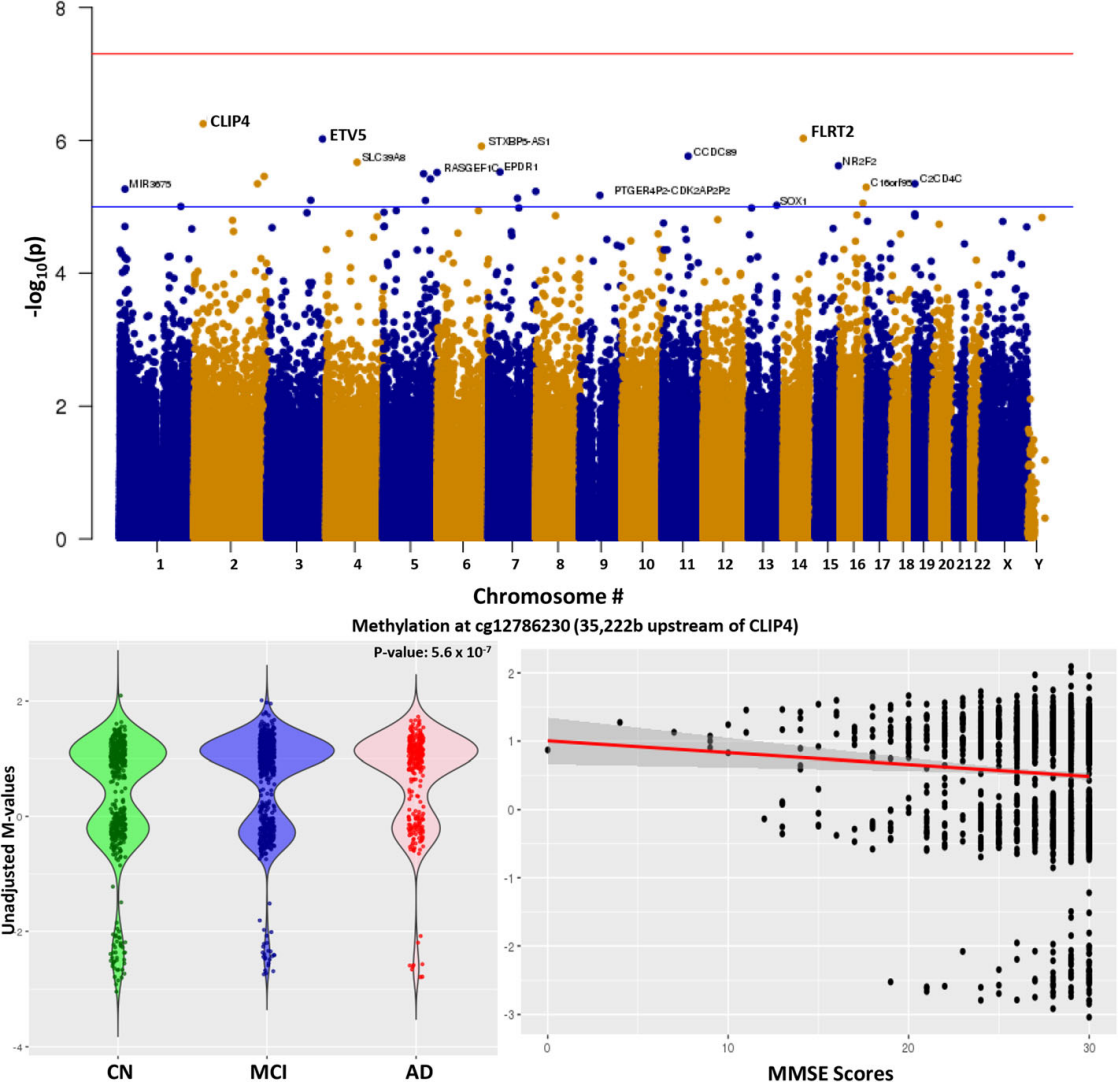
Comparison of DNA methylation in MCI (Mild cognitive impairment) vs CN (cognitively normal). **a** Manhattan plot showing the top hits in the MCI vs CN comparison. The blue line indicates *p* value threshold of 1 × 10^−5^ and the red line indicates *p* value threshold of 1 × 10^−7^. **b** Distribution of unadjusted *M* values in *CLIP4*, the top DMP across CN (green), MCI (blue), and AD (red). Violin plots outline the spread of the data. **c** Correlation of MMSE scores with differential methylation at the *CLIP4* locus.

In a similar way, we identified differential methylation from the MCI vs CN comparison, which yielded 25 DMPs at a *p* value threshold of 1 × 10^−5^ (Fig. 3a). The DMP that had the strongest association with MCI vs CN was annotated to *CLIP4* (Fig. 3b). The clustering of the methylation signal correlates with the presence of a SNP at the CpG or within the probe that appears to differentially correlate with disease status. *CLIP4* is a member of the CAP-Gly Domain Containing Linker Protein Family, an important paralog of which, CLIP3, is associated with microtubule binding. Again, TSEA analysis identified enrichment of the brain-specific signals (*P*_adj._-value = 0.0007). There was also a significant (*p* value = 2.0 × 10^−5^) correlation of MMSE score with DNA methylation differences at this locus (Fig. 3c). We also found neurogenesis, cell projection, and brain-specific high CpG-rich promoters as some of the most highly enriched pathways/components when the MCI vs. CN DMPs were annotated (Table 2).

**Fig. 4:**
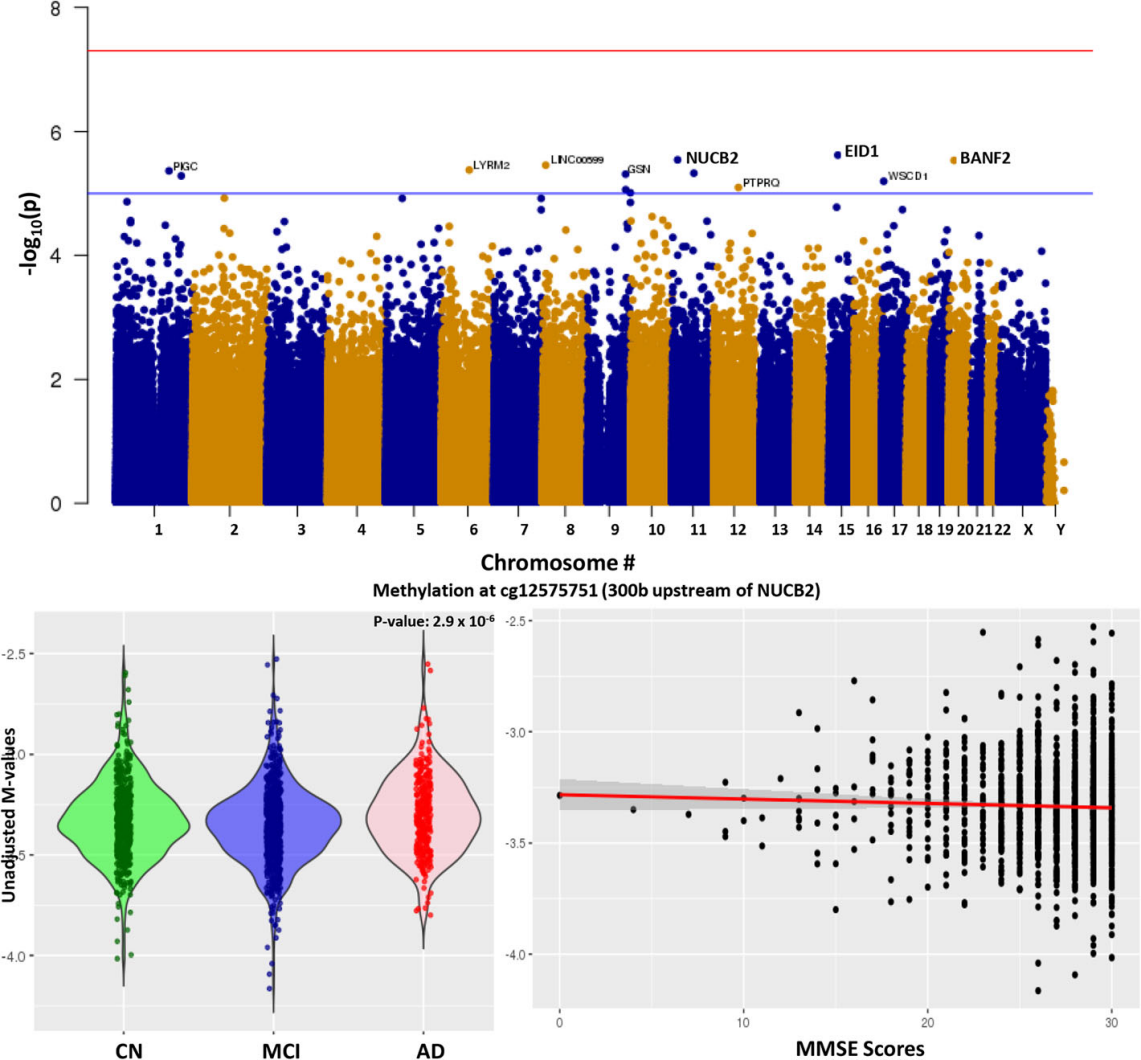
Comparison of DNA methylation in AD (Alzheimer’s disease) vs MCI (mild cognitive impairment). **a** Manhattan plot showing the top hits in the AD vs MCI comparison. The blue line indicates *p* value threshold of 1 × 10^−5^ and the red line indicates *p* value threshold of 1 × 10^−7^. **b** Distribution of unadjusted *M* values in *NUCB2*, the top DMP across CN (green), MCI (blue), and AD (red). Violin plots outline the spread of the data. **c** Correlation of MMSE scores with differential methylation at the *NUCB2* locus

Differential methylation analysis of the AD vs MCI comparison yielded 13 DMPs that were significant (Fig. 4a). The strongest associated DMP was annotated to *NUCB2* (nucleobindin 2), a calcium ion binding protein that regulates intracellular calcium levels. Given the small number of hits, TSEA showed no enrichment of brain-specific pathways, but a slight enrichment of lung-related pathways (Figure S3C, Table S3). There was a significant (*p* value = 4 × 10^−4^) correlation of MMSE score with DNA methylation differences at this locus (Fig. 4c). Interestingly, parallel testing in gene ontology analysis showed enrichment of genes that are downregulated in Alzheimer’s disease as well as cell projections, and neuronal pathways (Table 2). In addition, *BIN1*, *BDNF*, and *APOC1* while not the top most differentially methylated hits, were among the significant DMP hits (Figure S4A–C).

### Deriving genetic information from differential DNA methylation signals

Several studies have found an association of genetic variants with the DNA methylation signals at specific probes [22, 23]. To further evaluate the likelihood of DMPs correlating with AD, we also queried all the DMP-associated genes within the GWAS catalog for AD, (https://ebi.ac.uk/gwas/) which includes 72 individual GWAS studies and found overlaps between the GWAS hits and the DMPs from AD vs. CN, AD vs. MCI, and MCI vs. CN comparisons (Figure S5A). Some of the overlaps included *BIN1* (Figure S5C), *KCNN2* (Figure S5B), *DIP2C* (Figure S5C), *PAK2* (Figure S5D), *C3orf67* (Figure S5E), and *WNT3* (Figure S5F). We were also able to utilize the methylation data to identify disease-specific associations with some novel SNPs previously linked to neurodevelopmental and neuropsychiatric disorders. For example, *ANK3* has been associated with mental retardation, *SLC45A1* with intellectual developmental disorder, and *CHI3L1* with schizophrenia (Table S4), suggesting that differential methylation data may help reveal novel genetic variations that associate with AD. These associations could be interesting hypotheses requiring further testing.

### Replication of differential methylation signals across multiple datasets

Finally, we queried a second dataset for differential methylation at the loci identified in our study. A comprehensive study of about 1628 samples assessed human samples across several different types of tissues, including leukocytes, brain regions, and several cancer tissues [24]. Comparison of differential methylation in leukocytes from 65 healthy control subjects that were age 65 years or older with 35 AD subjects within the aforementioned study identified several DMPs. In an effort to replicate our findings from ADNI peripheral blood, we tested for overlaps across our study and the output from the above and observed overlaps across 11 CpGs (Table 3).

**Table 3:** Replication of differential methylation across datasets (using Fernandez et al. Leukocyte data)

Comparison	Gene	Discovery CpG	Discovery P-value	Replication CpG locus	Replication P-value
AD vs CN	ADCYAP1	cg16288125	3.02 × 10^-5^	chr18:905,549-905,550	3.78E-09
	EPHB3	cg22462726	2.57 × 10^-6^	chr3:184,561,230-184,561,231	1.78E-05
	GDF10	cg00414835	2.5 × 10^-5^	chr10:47,300,094-47,300,095	7.89E-04
	PARP1	cg27113848	2.74 × 10^-5^	chr1:226,408,700-226,408,701	1.25E-01
MCI vs AD	DAB2IP	cg20416296	8.69 × 10^-6^	chr9:121,698,699-121,698,700	4.49E-01
	MET	cg04432493	8.6 × 10^-5^	chr7:116,672,738-116,672,739	7.05E-03
	APOC1	cg07773593	6.06 × 10^-5^	chr19:44,914,258-44,914,259	9.97E-03
	ITGB1	cg05376034	6.42 × 10^-5^	chr10:32,958,721-32,958,722	6.20E-02
MCI vs CN	SOX1	cg07911664	9.5 × 10^-6^	chr13:112,066,581-112,066,582	1.12E-01
	WNT1	cg22376688	1.56 × 10^-5^	chr12:48,972,583-48,972,584	3.90E-06
	DAPK1	cg10240127	3.1 × 10-5	chr9:87,497,927-87,497,928	8.85E-04

## Discussion

We have successfully assayed peripheral blood samples from ADNI to investigate differential DNA methylation in mild cognitively impaired and Alzheimer’s disease patients across serial visits using the Illumina EPIC chip. The success rate for the experiment was 99.7%, with only 15 samples out of the total 1920 that failed the run and/or quality control thresholds. Our work establishes the robustness of DNA methylation as a peripheral marker and demonstrates the consistency and reproducibility of its detection at > 99% concordance across replicates.

The cross-diagnosis analysis demonstrates that a common set genomic loci in the periphery are differentially methylated in individuals with AD compared to normal healthy individuals. Several of these differential methylation marks were also replicated in a second peripheral DNA methylation dataset. Additionally, PB DNA methylation differences were found to be enriched near or within genes previously shown to associate with brain-associated pathways. The differential methylation at these sites correlates with cognitive scores, suggesting a relationship between the differential methylation with endophenotypes of disease progression.

When assessing the overlap in DNA methylation patterns in the periphery and the brain, previous studies have demonstrated that genome-wide DNA methylation profiles are specific to the tissue being studie d[16, 25–27]. These studies have suggested that even though many of the DMPs were associated with differentially expressed transcripts, blood-based epigenome-wide association studies from methylation arrays may not correlate with disease etiology [25]. In contrast, some other studies have shown conservation of DNA methylation patterns across blood and brain [18, 28, 29], specifically at promoter regions [18], or via co-expression modules that correlate the brain and the blood to age [29]. Our study picks up some signals in the periphery that are enriched for brain-specific loci; however, this warrants additional studies to detect the blood-brain overlap in DNA methylation. Interestingly, a recent article based on the ENIGMA studies (MRI readouts from 3337 individuals) demonstrated an association of blood DNA methylation with volumes of the hippocampus, thalamus, and nucleus accumbens (NAcc) [30].

The ADNI participant cohort has previously been used to identify novel biomarkers of disease development and progression [31–33], and is uniquely suited to measure and validate these changes. Ongoing work includes the integration of the methylation data with the rich phenotypic (e.g., cognitive, memory, neuroimaging) and multi-omic data (e.g., genotypic, expression, metabolomics) from the ADNI dataset. This will allow for the use of peripheral DNA methylation marks to function as a dynamic biomarker of disease progression and response to drug treatment.

Peripheral differential methylation has been used as a biomarker of disease occurrence and progression across several therapeutic areas including autoimmune diseases, cancers, and heart disease [34–36]. Previous methylation studies undertaken with PB or peripheral blood mononuclear cell (PBMC) samples mostly provided a snapshot of DNA methylation changes in the periphery that associated with disease status. A recent study described the identification of PB DNA methylation changes that associated with normal brain aging and cognitive decline in the Whitehall imaging study [37]. For most biomarkers being studied, longitudinal measures appear to more sensitively predict cognitive decline [38, 39]. Our study design includes longitudinal DNA samples and further analysis will measure dynamic changes in DNA methylation that associate with disease progression. The potential value of DMPs as a surrogate for disease is critically important and can change our approach to clinical studies. Presentation of these results gives the field an opportunity to further investigate and validate the DMPs as surrogates of disease.

## Methods

### Subjects

ADNI is a longitudinal study with approximately 50 sites across the USA and Canada that was launched in 2003 with a major goal being to track the AD progression using clinical and cognitive tests, magnetic resonance imaging (MRI), fludeoxyglucose PET, amyloid PET, cerebrospinal fluid, and blood biomarkers. The institutional review boards of all participating sites reviewed and approved the data collection protocol provided by ADNI. Clinical descriptions of the ADNI cohort have been published [40]. Six hundred and fifty-three individuals from two phases of ADNI (ADNI2 and ADNIGO) were selected for performing DNA methylation analysis (Table 1) based on the completeness of their other datasets (genotyping [APOE, TOMM40], genome wide array, whole genome sequencing, proteomic and imaging data). A total of 1720 samples were obtained, and randomized using a modified incomplete balanced block design, whereby all samples from a subject were on the same chip, with remaining chip space occupied by age-matched samples from a subject of the opposite sex with a different diagnosis. Unused chip space was leveraged for technical reproducibility assessment via replicated DNA samples. A total of 200 samples were replicated across all the chips (Figure S1), for a total 1920 samples processed. Among these replicates, we found consistent DNA methylation signals both within plates and across plates. The correlation coefficient was 99.63% when the replicates were on the same plate with the same scan date, and 99.25% when the replicates were on different plates with different scan dates (Table S1).

### EPIC chip runs

Illumina EPIC chips (Illumina, Inc., San Diego, CA, USA) were used to assay for DNA methylation levels according to published Illumina protocols. Genomic DNA samples obtained from NCRAD (National Centralized Repository for Alzheimer's Disease and Related Dementias) were bisulfite converted using the EZ-DNA Methylation kits (Zymo Research, Irvine, CA, USA) and subsequently analyzed using the Illumina Infinium HD methylation protocol on the HiScan (Illumina).

### Normalization and quality control methods

The derived beta values were transformed to *M* values and used for further analysis. The scan output was run through Genome Studio software (Illumina) to assay for initial QC metrics. One sample out of the total 1920 failed the run and had no CpG calls. The remaining samples had an average of CpG call of 864,640. Four additional samples failed quality control since ≥ 1% of CpG sites had a detection *p* value > 0.05 using watermelon [41]. All 1915 samples were normalized using the dasen method in wateRmelon [41].

### Sample identity checks

Sample sex was examined by computing the ratio of the X and Y probe intensities for each subject compared to their expected value, with > 99% of subjects mapping to the given sex (Figure S2A). The following R packages were used to check sample quality and possible sample mix-ups via sex-mismatches: Cham p[42], minfin [43], methylumi [44], and watermelon [41]. Additionally, we used the 59 tracking cpgs on the Illumina EPIC chips which are proxies for SNP fingerprinting (i.e., probe contains C allele that is a common variant), and compared those to the ADNI GWAS genotyping array data at the same positions (Figure S2B) using a clustering algorithm (*k* = 3) to convert cpg signal to genotype based on Hardy-Weinberg equilibrium. The GWAS data were procured from LONI (http://www.loni.usc.edu/). After normalization, quality control, and removal of duplicates, 1707 samples were analyzed for differences in DNA methylation.

### Statistical analysis

Since we wanted to include all the samples available for each subject in our initial analysis to compare across diagnoses, we fitted a mixed effects model on the *M* values to account for repeated measures of DNA methylation for the patients. This was done using the limma package [45–48] using dupcor estimated at the subject-level. We evaluated the association between DNA methylation level and diagnosis in multivariate models adjusted for age, sex, education, cell composition changes, and DNA storage/source in the model as shown in supplementary material. As it is known that peripheral blood cell composition can substantially affect methylation differences [41] between individuals, differential methylation analysis requires that any change in cell composition be adjusted for. Cell composition estimates were obtained using estimateCellCounts [43] at default settings such that estimates are made for CD8T, CD4T, NK, Bcell, Mono, and Gran. Because they lie in [0,1] and are constrained to sum to 1 within a sample, including all 6 values as covariates would induce multicollinearity. Therefore, only 5 cell type values are used as covariates. Furthermore, the difference in the storage of the sample used for DNA isolation (whole blood vs. buffy coat) had an impact on the cell composition, prompting its use as an additional covariate, as detailed in Supplementary Material.

### Functional analysis of top differentially methylated positions (DMPs)

Tissue specific analysis of differentially methylated marks was performed using Tissue Specific Expression Analysis (TSEA) at http://genetics.wustl.edu/jdlab/tsea/ [20]. Gene ontology analysis was performed using the molecular signature database (MSigDB) at http://software.broadinstitute.org/gsea/index.jsp [49, 50]. Curated gene sets (Biocarta, KEGG, and Reactome), Gene ontology gene sets (GO biological process, GO cellular component, and GO molecular function), and Immunologic signatures were included in the pathway analysis, and an FDR *q* value of 0.05 was set as the threshold.

Additional details regarding statistical analyses are included in supplemental information.

## Supplementary information

**Additional file 1: Fig. S1**. Plate layout after randomization. **Fig. S2**. Quality Control and Confirmation of Sample Identity. **Fig. S3**. Tissue-Specific Expression Analysis identifies Brain-Specific Enrichment. **Fig. S4**. DNA Methylation Differences in Genes Previously Associated with AD Pathogenesis. **Fig. S5**. GWAS Hits Show Concordant DNA Methylation differences. **Table S1**. Correlation of Replicate Samples (200 total technical replicates) within non-normalized data. **Table S2**. Table of all DMPs. **Table S3**. Interpretation of TSEA results.

## Data Availability

The datasets generated and analyzed in the current study are available in the LONI repository (http://www.loni.usc.edu/).

## Abbreviations

ADNI: Alzheimer’s Disease Neuroimaging Initiative
AD: Alzheimer’s disease
CN: Cognitively normal
CSF: Cerebrospinal fluid
DMP: Differentially methylated position
EWAS: Epigenome-wide association study
GWAS: Genome-wide association study
MCI: Mild cognitive impairment
MMSE: Mini Mental Status Examination
MRI: Magnetic resonance imaging
NCRAD: National Centralized Repository for Alzheimer's Disease and Related Dementias
NfL: Neurofilament light
OMIM: Online Mendelian Inheritance in Man
PB: Peripheral blood
PBMC: Peripheral blood mononuclear cells
SNP: Single nucleotide polymorphism
TSEA: Tissue Specific Expression Analysis
TSS: Transcriptional Start Site
UTR: Untranslated regions
