# Supplementary material for: Harnessing peripheral DNA methylation differences in the Alzheimer’s Disease Neuroimaging Initiative (ADNI) to reveal novel biomarkers of disease
Source: Clin Epigenetics. 2020 Jun 15;12:84. doi: 10.1186/s13148-020-00864-y (PMC7294637; doi:10.1186/s13148-020-00864-y)
Supplement: Supplementary file 1 — Additional file 1: Fig. S1. Plate layout after randomization. Fig. S2. Quality Control and Confirmation of Sample Identity. Fig. S3. Tissue-Specific Expression Analysis identifies Brain-Specific Enrichment. Fig. S4. DNA Methylation Differences in Genes Previously Associated with AD Pathogenesis. Fig. S5. GWAS Hits Show Concordant DNA Methylation differences. Table S1. Correlation of Replicate Samples (200 total technical replicates) within non-normalized data. Table S2. Table of all DMPs. Table S3. Interpretation of TSEA results. [file 13148_2020_864_MOESM1_ESM.docx]

**
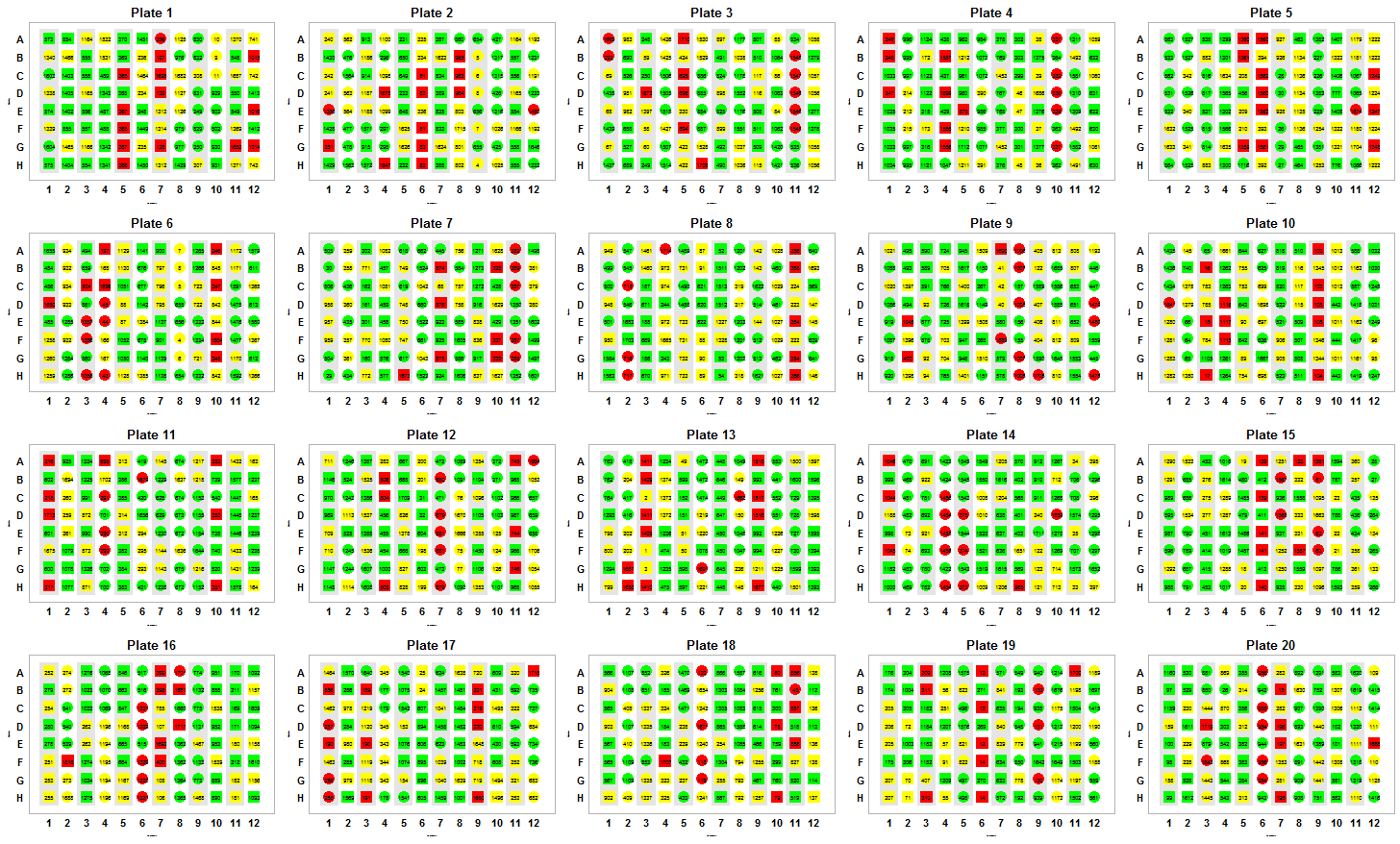
Fig. S1**.

**Fig. S1. Plate layout after randomization.** 1920 Samples were accommodated on 20 x 96-well plates after randomizing using the incomplete modified balanced block design using Individual age and gender as covariates. All longitudinal samples from a single subject were included on the same chip to avoid batch effects introduced by chip runs. Red = AD, Green = MCI, Yellow = CN. Circle = Females, Squares = Males


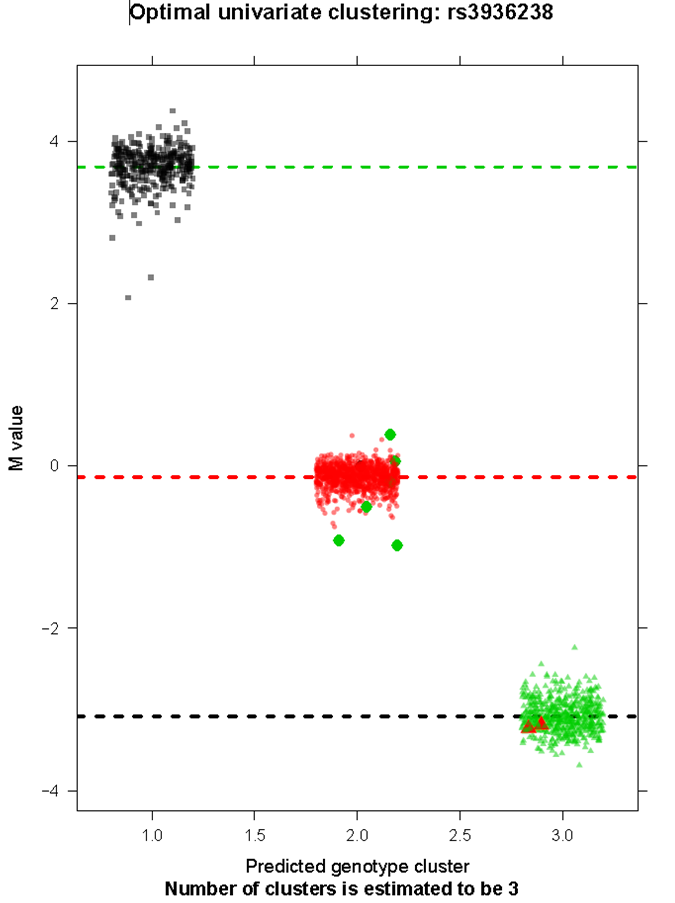
**Fig. S2.**

**B.**

**A.**

**
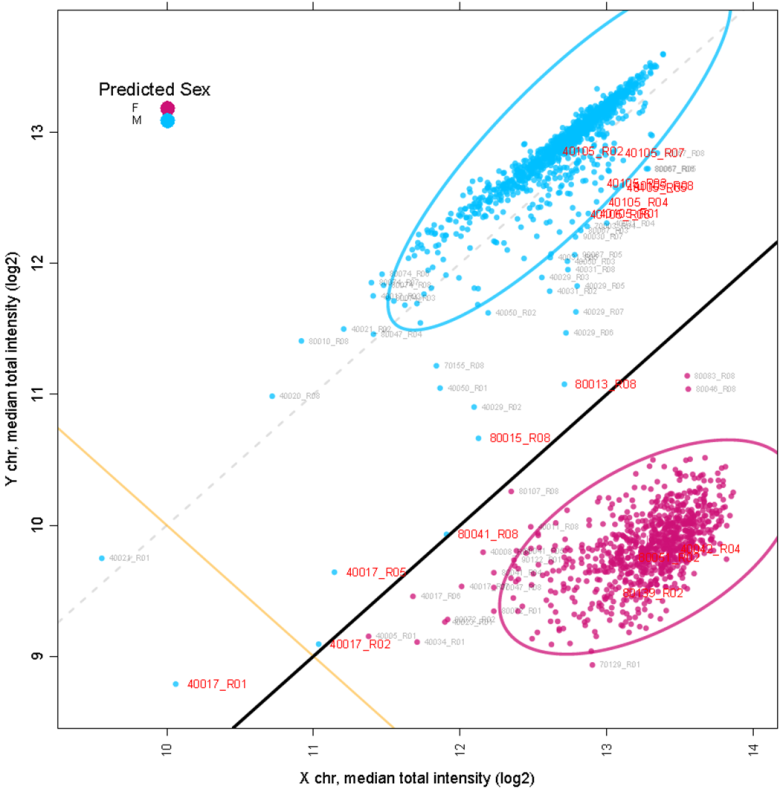
**

**Fig. S2. Quality Control and Confirmation of Sample Identity.** All samples that cleared QC thresholds were subjected to (A) Sex checks, and (B) Identity checks. (A) Sex checks were performed using median signal intensities from probes situated on X- and Y- chromosomes and using this to differentiate males from females. To ascertain the amount of uncertainty in the predictions, 95% prediction ellipses based on the predicted coordinates were computed and plotted. Those with predicted mismatches are shown in red font with the last digits of their array ID, and most come from two arrays with poor quality overall. Samples falling below a line perpendicular to the identity line at the point (10,10) are also considered suspect based on weak total intensity (salmon line). (B) GWAS data from the subjects used for methylation analyses were correlated with the M-value output from the 59 SNPs included in the EPIC chip to identify subjects. Individual clusters in panel B indicate individual genotypes. Output from both the QC/identity checks helped determine samples to be discarded from the analyses.**
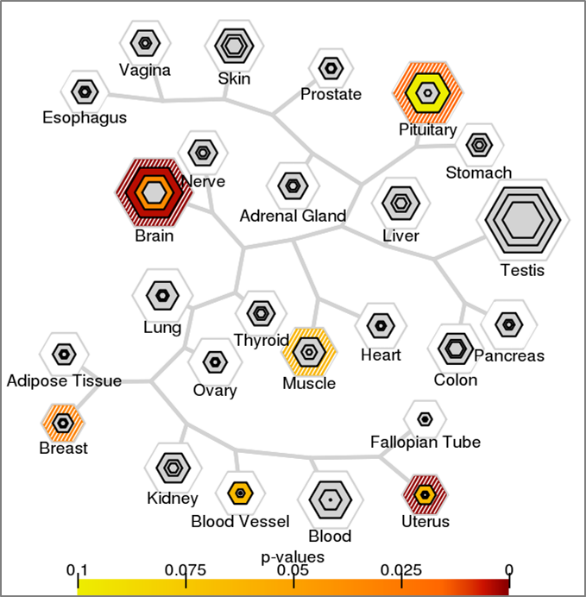
 Fig. S3.**

**A.**

**
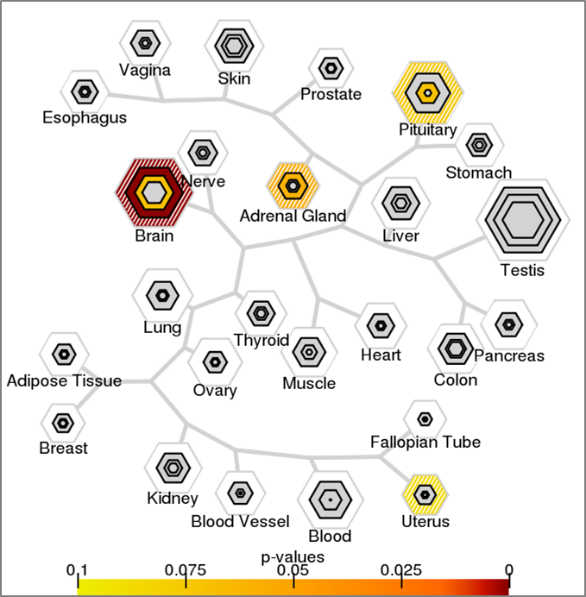
Fig. S3. Tissue-Specific Expression Analysis identifies Brain-Specific Enrichment**. The top hits from the methylation analysis for each comparison: (A) AD vs CN, (B) MCI vs CN, (C) AD vs MCI was entered into the TSEA console which uses GTex data to identify the tissue from which specific expression signals are derived. A 50kb distance to the TSS was used as the cutoff to identify specific genes to be considered. The AD vs CN and MCI vs CN comparisons both identified brain as the most highly represented tissue in these analyses. For all these analyses, transcripts from the processed GTEx transcripts that are specifically expressed or enriched have been identified by calculating the Specificity Index thresholds (pSI) of varying stringency[1,2]. For example, pSI smaller than .01 identifies a larger number of relatively enriched transcripts, while those at pSI smaller than .0001 will be just the subset that is relatively specific. For each cell type, the size of the bullseye is scaled to the number of specific and enriched transcripts at different stringency thresholds. Bullseyes are color coded by Fisher’s exact test p values as shown.

**C.**

**B.**

**
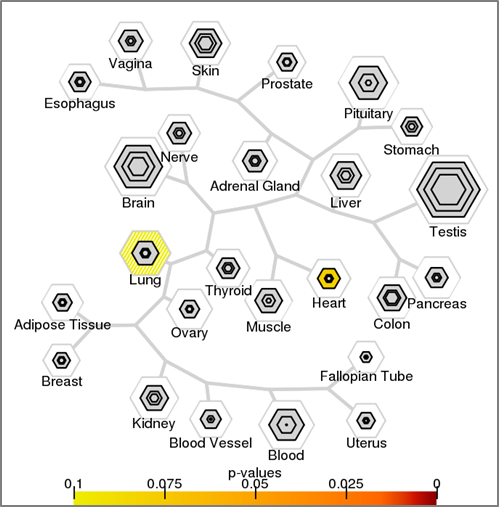
**

**
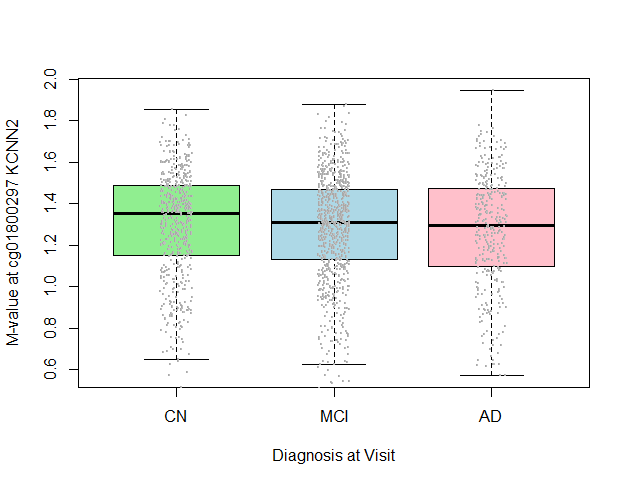

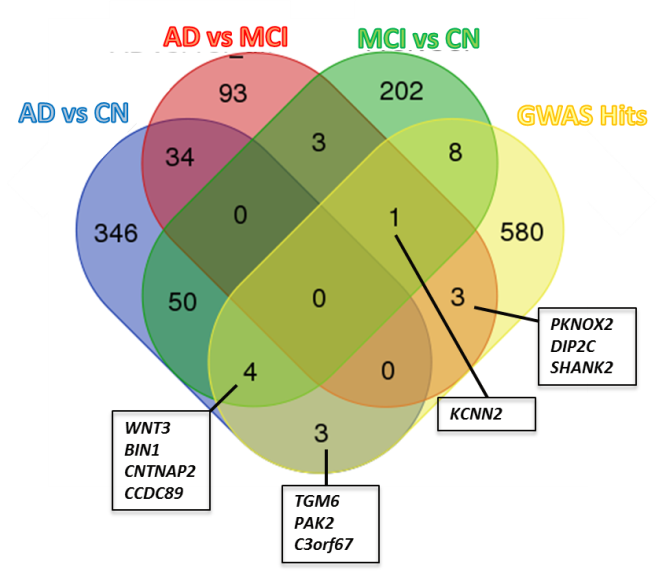
Fig. S4.**

**B.**

**A.**

**
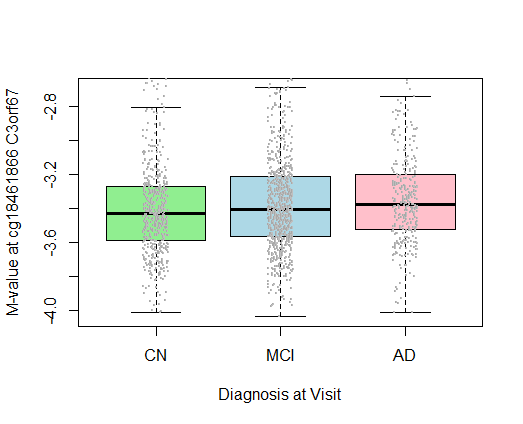

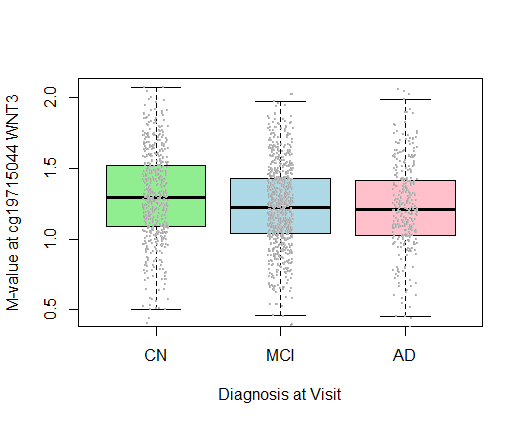

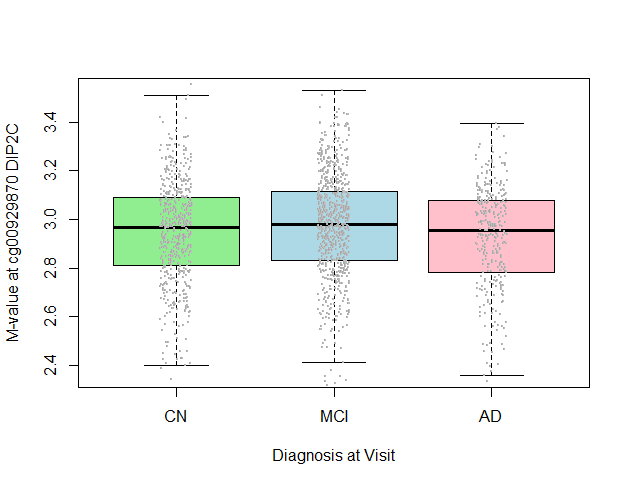
**

**
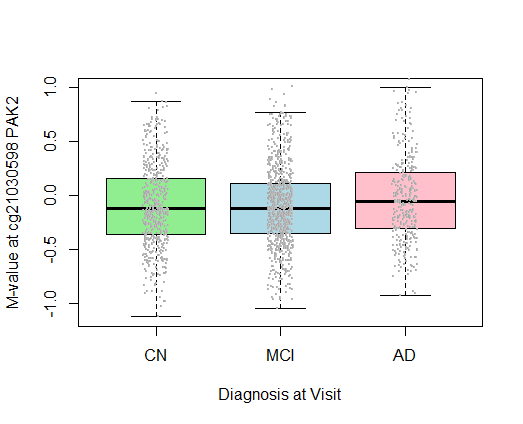
**

**Fig. S4. GWAS Hits Show Concordant DNA Methylation Changes.**  Several of the differentially methylated positions were within 50kb of a GWAS hit (Fig. 3). The distributions of the unadjusted M-values are shown within each diagnosis group.

**Table S1**. Correlation of Replicate Samples (200 total technical replicates) within non-normalized data

|  | Pearson Correlation | SD | 95% CI |
| --- | --- | --- | --- |
| Mean within subject | 99.42 | 1.2% | 97.99-99.84 |
| Replicates on same plate with same scan date | 99.63 | 0.32% |  |
| Replicates on different plates with different scan dates | 99.25% | 1.8% |  |

**Table S2. Table of all DMPs (excel sheet)**

**Table S3. Interpretation of TSEA results** [1,2]

|  | **AD vs CN** | | | | | | |
| --- | --- | --- | --- | --- | --- | --- | --- |
| **Tissues and P-Values** | **0.05** | **0.01** | | | **0.001** | **0.0001** | |
| Adipose Tissue | 0.11 ( 0.251 ) | 0.577 ( 0.869 ) | | | 1.0 ( 1.0 ) | 1.0 ( 1.0 ) | |
| Adrenal Gland | 0.592 ( 0.744 ) | 0.832 ( 0.977 ) | | | 1.0 ( 1.0 ) | 1.0 ( 1.0 ) | |
| Blood | 0.724 ( 0.811 ) | 0.774 ( 0.977 ) | | | 1.0 ( 1.0 ) | 1.0 ( 1.0 ) | |
| Blood Vessel | 0.197 ( 0.379 ) | 0.007 ( 0.064 ) | | | 0.259 ( 0.741 ) | 1.0 ( 1.0 ) | |
| Brain | 7.839e-05 ( 0.0009799 ) | 0.0001814 ( 0.005 ) | | | 0.001 ( 0.035 ) | 0.064 ( 0.799 ) | |
| Breast | 0.005 ( 0.031 ) | 0.207 ( 0.516 ) | | | 1.0 ( 1.0 ) | 1.0 ( 1.0 ) | |
| Colon | 0.275 ( 0.458 ) | 0.938 ( 0.977 ) | | | 0.787 ( 1.0 ) | 0.593 ( 1.0 ) | |
| Esophagus | 0.625 ( 0.744 ) | 0.923 ( 0.977 ) | | | 0.413 ( 0.794 ) | 0.19 ( 0.86 ) | |
| Fallopian Tube | 0.072 ( 0.225 ) | 0.525 ( 0.869 ) | | | 1.0 ( 1.0 ) | 1.0 ( 1.0 ) | |
| Heart | 0.401 ( 0.59 ) | 0.278 ( 0.631 ) | | | 0.329 ( 0.769 ) | 0.144 ( 0.86 ) | |
| Kidney | 0.919 ( 0.919 ) | 0.591 ( 0.869 ) | | | 0.338 ( 0.769 ) | 0.344 ( 0.956 ) | |
| Liver | 0.891 ( 0.919 ) | 0.884 ( 0.977 ) | | | 0.759 ( 1.0 ) | 1.0 ( 1.0 ) | |
| Lung | 0.598 ( 0.744 ) | 0.721 ( 0.977 ) | | | 1.0 ( 1.0 ) | 1.0 ( 1.0 ) | |
| Muscle | 0.012 ( 0.062 ) | 0.042 ( 0.194 ) | | | 0.602 ( 1.0 ) | 0.134 ( 0.86 ) | |
| Nerve | 0.128 ( 0.267 ) | 0.089 ( 0.254 ) | | | 0.034 ( 0.28 ) | 0.216 ( 0.86 ) | |
| Ovary | 0.042 ( 0.151 ) | 0.054 ( 0.194 ) | | | 0.046 ( 0.285 ) | 0.013 ( 0.328 ) | |
| Pancreas | 0.275 ( 0.458 ) | 0.05 ( 0.194 ) | | | 0.379 ( 0.79 ) | 1.0 ( 1.0 ) | |
| Pituitary | 0.002 ( 0.016 ) | 0.016 ( 0.099 ) | | | 0.14 ( 0.583 ) | 1.0 ( 1.0 ) | |
| Prostate | 0.746 ( 0.811 ) | 0.352 ( 0.733 ) | | | 1.0 ( 1.0 ) | 1.0 ( 1.0 ) | |
| Skin | 0.401 ( 0.59 ) | 0.791 ( 0.977 ) | | | 0.907 ( 1.0 ) | 0.678 ( 1.0 ) | |
| Stomach | 0.102 ( 0.251 ) | 1.0 ( 1.0 ) | | | 1.0 ( 1.0 ) | 1.0 ( 1.0 ) | |
| Testis | 0.436 ( 0.605 ) | 0.398 ( 0.766 ) | | | 0.267 ( 0.741 ) | 0.274 ( 0.86 ) | |
| Thyroid | 0.105 ( 0.251 ) | 0.53 ( 0.869 ) | | | 0.18 ( 0.644 ) | 0.275 ( 0.86 ) | |
| Uterus | 3.856e-05 ( 0.0009639 ) | 0.008 ( 0.064 ) | | | 0.022 ( 0.276 ) | 1.0 ( 1.0 ) | |
| Vagina | 0.03 ( 0.125 ) | 0.091 ( 0.254 ) | | | 0.105 ( 0.525 ) | 1.0 ( 1.0 ) | |
|  | **MCI vs CN** | | | | | | |
| **Tissues and P-Values** | **0.05** | | **0.01** | **0.001** | | | **0.0001** |
| Adipose Tissue | 0.438 ( 0.608 ) | | 0.612 ( 1.0 ) | 1.0 ( 1.0 ) | | | 1.0 ( 1.0 ) |
| Adrenal Gland | 0.004 ( 0.056 ) | | 0.005 ( 0.059 ) | 0.046 ( 0.387 ) | | | 1.0 ( 1.0 ) |
| Blood | 0.986 ( 0.986 ) | | 0.959 ( 1.0 ) | 1.0 ( 1.0 ) | | | 1.0 ( 1.0 ) |
| Blood Vessel | 0.824 ( 0.896 ) | | 0.667 ( 1.0 ) | 1.0 ( 1.0 ) | | | 1.0 ( 1.0 ) |
| Brain | 2.902e-05 ( 0.0007256 ) | | 4.855e-06 ( 0.0001214 ) | 0.006 ( 0.07 ) | | | 0.28 ( 1.0 ) |
| Breast | 0.26 ( 0.498 ) | | 1.0 ( 1.0 ) | 1.0 ( 1.0 ) | | | 1.0 ( 1.0 ) |
| Colon | 0.339 ( 0.498 ) | | 0.367 ( 0.918 ) | 0.528 ( 1.0 ) | | | 0.354 ( 1.0 ) |
| Esophagus | 0.603 ( 0.779 ) | | 0.713 ( 1.0 ) | 1.0 ( 1.0 ) | | | 1.0 ( 1.0 ) |
| Fallopian Tube | 0.226 ( 0.47 ) | | 1.0 ( 1.0 ) | 1.0 ( 1.0 ) | | | 1.0 ( 1.0 ) |
| Heart | 0.715 ( 0.846 ) | | 1.0 ( 1.0 ) | 1.0 ( 1.0 ) | | | 1.0 ( 1.0 ) |
| Kidney | 0.068 ( 0.323 ) | | 0.869 ( 1.0 ) | 0.445 ( 1.0 ) | | | 0.185 ( 1.0 ) |
| Liver | 0.744 ( 0.846 ) | | 0.709 ( 1.0 ) | 1.0 ( 1.0 ) | | | 1.0 ( 1.0 ) |
| Lung | 0.903 ( 0.94 ) | | 0.84 ( 1.0 ) | 1.0 ( 1.0 ) | | | 1.0 ( 1.0 ) |
| Muscle | 0.204 ( 0.463 ) | | 0.121 ( 0.44 ) | 0.361 ( 1.0 ) | | | 0.068 ( 1.0 ) |
| Nerve | 0.077 ( 0.323 ) | | 0.19 ( 0.526 ) | 0.292 ( 0.992 ) | | | 1.0 ( 1.0 ) |
| Ovary | 0.154 ( 0.463 ) | | 0.078 ( 0.389 ) | 0.154 ( 0.962 ) | | | 0.083 ( 1.0 ) |
| Pancreas | 0.339 ( 0.498 ) | | 1.0 ( 1.0 ) | 1.0 ( 1.0 ) | | | 1.0 ( 1.0 ) |
| Pituitary | 0.009 ( 0.074 ) | | 0.067 ( 0.389 ) | 0.003 ( 0.07 ) | | | 1.0 ( 1.0 ) |
| Prostate | 0.286 ( 0.498 ) | | 0.645 ( 1.0 ) | 1.0 ( 1.0 ) | | | 1.0 ( 1.0 ) |
| Skin | 0.17 ( 0.463 ) | | 0.048 ( 0.389 ) | 0.318 ( 0.992 ) | | | 0.424 ( 1.0 ) |
| Stomach | 0.623 ( 0.779 ) | | 1.0 ( 1.0 ) | 1.0 ( 1.0 ) | | | 1.0 ( 1.0 ) |
| Testis | 0.198 ( 0.463 ) | | 0.123 ( 0.44 ) | 0.263 ( 0.992 ) | | | 1.0 ( 1.0 ) |
| Thyroid | 0.099 ( 0.352 ) | | 0.155 ( 0.485 ) | 0.315 ( 0.992 ) | | | 1.0 ( 1.0 ) |
| Uterus | 0.014 ( 0.087 ) | | 0.57 ( 1.0 ) | 1.0 ( 1.0 ) | | | 1.0 ( 1.0 ) |
| Vagina | 0.321 ( 0.498 ) | | 0.447 ( 1.0 ) | 1.0 ( 1.0 ) | | | 1.0 ( 1.0 ) |
|  | **AD vs MCI** | | | | | | |
| **Tissues and P-Values** | **0.05** | | **0.01** | **0.001** | | | **0.0001** |
| Adipose Tissue | 0.236 ( 0.54 ) | | 1.0 ( 1.0 ) | 1.0 ( 1.0 ) | | | 1.0 ( 1.0 ) |
| Adrenal Gland | 0.542 ( 0.753 ) | | 0.334 ( 1.0 ) | 0.231 ( 1.0 ) | | | 1.0 ( 1.0 ) |
| Blood | 0.376 ( 0.626 ) | | 0.217 ( 1.0 ) | 0.494 ( 1.0 ) | | | 1.0 ( 1.0 ) |
| Blood Vessel | 0.247 ( 0.54 ) | | 1.0 ( 1.0 ) | 1.0 ( 1.0 ) | | | 1.0 ( 1.0 ) |
| Brain | 0.021 ( 0.176 ) | | 0.034 ( 0.287 ) | 0.151 ( 1.0 ) | | | 0.551 ( 1.0 ) |
| Breast | 0.742 ( 0.844 ) | | 1.0 ( 1.0 ) | 1.0 ( 1.0 ) | | | 1.0 ( 1.0 ) |
| Colon | 0.935 ( 0.935 ) | | 1.0 ( 1.0 ) | 1.0 ( 1.0 ) | | | 1.0 ( 1.0 ) |
| Esophagus | 0.833 ( 0.898 ) | | 0.243 ( 1.0 ) | 0.179 ( 1.0 ) | | | 0.075 ( 1.0 ) |
| Fallopian Tube | 0.453 ( 0.708 ) | | 1.0 ( 1.0 ) | 1.0 ( 1.0 ) | | | 1.0 ( 1.0 ) |
| Heart | 0.011 ( 0.134 ) | | 0.003 ( 0.079 ) | 1.0 ( 1.0 ) | | | 1.0 ( 1.0 ) |
| Kidney | 0.159 ( 0.54 ) | | 0.454 ( 1.0 ) | 1.0 ( 1.0 ) | | | 1.0 ( 1.0 ) |
| Liver | 0.328 ( 0.586 ) | | 0.851 ( 1.0 ) | 1.0 ( 1.0 ) | | | 1.0 ( 1.0 ) |
| Lung | 0.004 ( 0.099 ) | | 0.16 ( 1.0 ) | 1.0 ( 1.0 ) | | | 1.0 ( 1.0 ) |
| Muscle | 0.261 ( 0.54 ) | | 0.425 ( 1.0 ) | 0.289 ( 1.0 ) | | | 1.0 ( 1.0 ) |
| Nerve | 0.252 ( 0.54 ) | | 1.0 ( 1.0 ) | 1.0 ( 1.0 ) | | | 1.0 ( 1.0 ) |
| Ovary | 0.573 ( 0.754 ) | | 1.0 ( 1.0 ) | 1.0 ( 1.0 ) | | | 1.0 ( 1.0 ) |
| Pancreas | 0.264 ( 0.54 ) | | 0.629 ( 1.0 ) | 1.0 ( 1.0 ) | | | 1.0 ( 1.0 ) |
| Pituitary | 0.044 ( 0.272 ) | | 0.019 ( 0.24 ) | 0.17 ( 1.0 ) | | | 1.0 ( 1.0 ) |
| Prostate | 0.516 ( 0.753 ) | | 1.0 ( 1.0 ) | 1.0 ( 1.0 ) | | | 1.0 ( 1.0 ) |
| Skin | 0.862 ( 0.898 ) | | 0.599 ( 1.0 ) | 0.585 ( 1.0 ) | | | 1.0 ( 1.0 ) |
| Stomach | 0.638 ( 0.775 ) | | 0.592 ( 1.0 ) | 0.247 ( 1.0 ) | | | 1.0 ( 1.0 ) |
| Testis | 0.651 ( 0.775 ) | | 0.652 ( 1.0 ) | 0.498 ( 1.0 ) | | | 0.671 ( 1.0 ) |
| Thyroid | 0.149 ( 0.54 ) | | 0.649 ( 1.0 ) | 1.0 ( 1.0 ) | | | 1.0 ( 1.0 ) |
| Uterus | 0.07 ( 0.352 ) | | 0.475 ( 1.0 ) | 1.0 ( 1.0 ) | | | 1.0 ( 1.0 ) |
| Vagina | 0.281 ( 0.54 ) | | 0.32 ( 1.0 ) | 1.0 ( 1.0 ) | | | 1.0 ( 1.0 ) |

* The values in the tables are the Fisher's Exact p-values, followed by the Benjamini-Hochberg (BH) corrected values in parentheses.

**Table S4. Examples of SNPs Revealed by DNA Methylation Data to Associate with Disease Status**

| **Probe Name** | **SNP rsID/ MAF** | **Nearest Gene** | **Distance from TSS** | **Associated Pathways/ Ontology Terms** |
| --- | --- | --- | --- | --- |
| cg14144366 | rs1241524/ 0.18 | ANK3 | 22028 | Golgi transport; protein metabolism; structural constituent of cytoskeleton |
| cg24446235 | rs585883/ 0.07 | ATP2B2 | 14894 | Cardiac conduction; response to elevated platelet cytosolic Ca^2+^; calcium ion binding; calmodulin binding |
| cg17014757 | rs1039993/ 0.28 | CHI3L1 | 174 | Innate immune system; carbohydrate binding and hydrolase activity; hydrolysis of O-glycosyl compounds. |
| cg06378561 | rs9854823/ 0.24 | FSTL1 | 18633 | Signaling by GPCR; calcium ion binding; heparin binding |
| cg00034101 | rs1148944/ 0.21 | HEYL | 75 | Notch signaling; PI3K-Akt signaling; DNA binding transcription factor activity |
| cg03084184 | rs4880338/ 0.40 | JAKMIP3 | 223 | microtubule binding; kinase binding |
| cg02823329 | rs1695770/ 0.25 | PIK3R5 | 21097 | Apoptotic pathways; cardiac hypertrophy; G-protein beta/gamma-subunit complex binding; 1-phosphatidylinositol-3-kinase regulator activity |
| cg13089904 | rs17032406/ 0.06 | SLC45A1 | 41117 | symporter activity |

**MAF- minor allele frequency (obtained from dbSNP build 151))**

**Supplementary Methods**

**Subjects:** ADNI is a longitudinal study with approximately 50 sites across the United States and Canada that was launched in 2003 with a major goal being to track the AD progression using clinical and cognitive tests, magnetic resonance imaging (MRI), fludeoxyglucose PET, amyloid PET, cerebrospinal fluid, and blood biomarkers. The institutional review boards of all participating sites reviewed and approved the data collection protocol provided by ADNI. Six hundred and fifty-three individuals from two phases of ADNI (ADNI2 and ADNIGO) were selected for performing DNA methylation analysis. All subjects had genotyping (APOE, TOMM40), genome wide array, and whole genome sequencing, as well as transcriptomic array and complete CSF biomarker and imaging data available. Two experimental designs were used for patient selection: a longitudinal design (patients with samples at three individual visits), and converters design (patients converting from CN to MCI, CN to AD, or MCI to AD). The current study focuses on differential methylation analysis of subjects based on diagnosis, with the longitudinal analysis forming the basis for a future publication. A total of 1720 samples were obtained, and randomized using a modified incomplete balanced block design, whereby all samples from a subject were on the same chip, with remaining chip space occupied by age- and sex-matched samples from a subject with a different diagnosis. Unused chip space was leveraged for technical reproducibility assessment via replicated DNA samples. A total of 200 samples were replicated across all the chips (Figure S1).

*
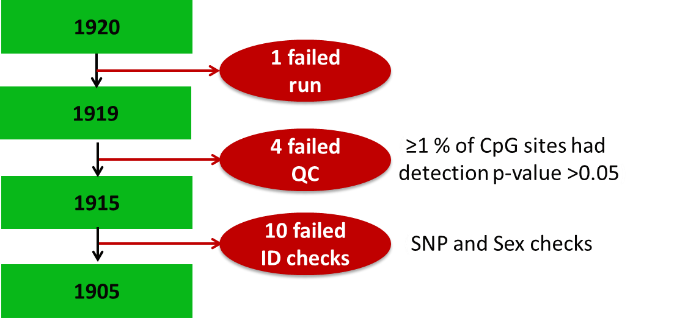
***Selection of final sample set**: As shown on the right, one sample out of the total 1920 failed the run and had no CpG calls. The remaining samples had an average of CpG call of 864,640. Four additional samples failed quality control since ≥1 % of CpG sites had a detection p-value >0.05. Of the remaining 1915 samples, 10 additional samples were removed from analysis due to chip-specific issues, identified from the sex/ID checks.

Among the replicates included, several decisions were used to pick the replicate used for the analysis.

- Whenever possible, all multi-sample sets would be analyzed from the same chip- samples would stay with their set
- If whole sample sets were replicated, and both sets of samples performed equally well, one of the sets was randomly chosen.
- If there were individual dropouts from a multi-sample set, the more complete set was used for analysis
- For individual samples placed on the same chip, random selection was done to select samples

**Normalization & Quality Control Methods:** The scan output was run through Genome Studio software (Illumina) to assay for initial QC metrics. The 1915 samples were normalized using the dasen method in WateRmelon: R/Bioconductor open source package according to Pidsley(2013).

**Sample Identity Checks:** Sample sex was examined by computing the ratio of the X and Y probe intensities for each subject compared to their expected value, with >99% of subjects mapping to the given sex. The fifty-nine probes targeting SNP sites on the Illumina EPIC chips were used for comparison to the GWAS data from the same subjects. The GWAS data were procured from LONI (<http://www.loni.usc.edu/>). After normalization, QC, and removal of duplicates, 1708 samples were analyzed for differences in DNA methylation.

**Statistical Analysis:** To retain the power of detecting small changes within the experiment, we included all the samples available for each subject in our initial analysis to compare across diagnoses. To account for repetition of an individual subjects, a mixed effects model was fitted to account for repeated measures of DNA methylation for the patients. However, since all samples from the same subject were ran on the same chip, correlation coefficients were on the high range (0.51), while using technical duplicates we saw that decrease to 0.21, so we decided to use the average of the two values (0.36) as our estimated correlation within subject. The association between DNA methylation level and diagnosis was analyzed in multivariate models adjusted for age, sex, education, cell composition changes, and DNA storage/source with multiple testing correction using Benjamini-Hochberg method, as detailed below.

Since peripheral blood cell composition can substantially affect methylation differences between individuals, differential methylation analysis requires that any change in cell composition be adjusted for. This was corrected by using a bioinformatic method published by Houseman et al. in which the individual cell type data are overlaid on the methylation data to be analyzed to de-convolute the impact of individual cell types.

Furthermore, the sample used for DNA isolation were found to be stored in various ways, with some of them being stored as whole blood for more than 24 hours before processing to buffy coat, and others being processed immediately upon receipt of samples. The storage and processing has been found to have an impact on the cell composition, prompting us to use DNA storage as an additional covariate.

The final model used was of the form:

model.matrix(~0 + DXatVisit + SEX + AgeatDrawVisit + education + smoke

Gran +CD4T +NK +Bcell +Mono + buffy+

Gran*buffy +CD4T*buffy +NK*buffy +Bcell*buffy +Mono*buffy).

Here, DXatVisit is a categorical variable (factor) denoting the cognitive status (CN, MCI, AD) of the subject at a given time point during which the sample was obtained and AgeatDrawVisit was the subject’s age at that timepoint. Education was a numeric variable (years of education) as reported in the ADNIMERGE R package, smoking was a binary indicator variable, and sex was a two-level factor. Buffy was an indicator binary variable to denote how the sample was processed by ADNI, as described in previous paragraph, and this was used to form interaction terms with the cell type, as it is expected that buffy coat samples differ in the cellular composition.

Based on the parameter estimates obtained from this model, contrasts were tested using the eBayes function with robust = TRUE in the limma package.

**Functional Analysis of Top Differentially Methylated Positions (DMPs):** Tissue specific analysis of differentially methylated marks was performed using Tissue Specific Expression Analysis (TSEA) at <http://genetics.wustl.edu/jdlab/tsea/>. Gene ontology analysis was performed using the molecular signature database (MSigDB) at <http://software.broadinstitute.org/gsea/index.jsp>. Curated gene sets (Biocarta, KEGG, and Reactome), Gene ontology gene sets (GO biological process, GO cellular component, and GO molecular function), and Immunologic signatures were included in the pathway analysis, and an FDR q-value of 0.05 was set as the threshold.

**Supplementary References**

1. Dougherty JD, Schmidt EF, Nakajima M, Heintz N. Analytical approaches to RNA profiling data for the identification of genes enriched in specific cells. Nucleic Acids Res. 2010;38: 4218–4230. doi:10.1093/nar/gkq130

2. Xu X, Wells AB, O’Brien DR, Nehorai A, Dougherty JD. Cell Type-Specific Expression Analysis to Identify Putative Cellular Mechanisms for Neurogenetic Disorders. J Neurosci. 2014;34: 1420–1431. doi:10.1523/JNEUROSCI.4488-13.2014

3. Wang M, Beckmann ND, Roussos P, Wang E, Zhou X, Wang Q, et al. The Mount Sinai cohort of large-scale genomic, transcriptomic and proteomic data in Alzheimer’s disease. Sci Data. 2018;5: 180185. doi:10.1038/sdata.2018.185

4. Hodes RJ, Buckholtz N. Accelerating Medicines Partnership: Alzheimer’s Disease (AMP-AD) Knowledge Portal Aids Alzheimer’s Drug Discovery through Open Data Sharing. Expert Opin Ther Targets. Taylor & Francis; 2016;20: 389–391. doi:10.1517/14728222.2016.1135132.

5. Houseman EA, Molitor J, Marsit CJ. Reference-free cell mixture adjustments in analysis of DNA methylation data. Bioinformatics. 2014;30(10):1431-439.
